# Polymeric prosthetic heart valves: A review of current technologies and future directions

**DOI:** 10.3389/fcvm.2023.1137827

**Published:** 2023-03-09

**Authors:** Sameer K. Singh, Mateusz Kachel, Estibaliz Castillero, Yingfei Xue, David Kalfa, Giovanni Ferrari, Isaac George

**Affiliations:** ^1^Division of Cardiothoracic Surgery, New York Presbyterian Hospital, College of Physicians and Surgeons of Columbia University, New York, NY, United States; ^2^Cardiovascular Research Foundation, New York, NY, United States; ^3^American Heart of Poland, Center for Cardiovascular Research and Development, Katowice, Poland

**Keywords:** polymer valve, polymer, heart valve, transcatheter and surgical aortic valve replacement, aortic valve

## Abstract

Valvular heart disease is an important source of cardiovascular morbidity and mortality. Current prosthetic valve replacement options, such as bioprosthetic and mechanical heart valves are limited by structural valve degeneration requiring reoperation or the need for lifelong anticoagulation. Several new polymer technologies have been developed in recent years in the hope of creating an ideal polymeric heart valve substitute that overcomes these limitations. These compounds and valve devices are in various stages of research and development and have unique strengths and limitations inherent to their properties. This review summarizes the current literature available for the latest polymer heart valve technologies and compares important characteristics necessary for a successful valve replacement therapy, including hydrodynamic performance, thrombogenicity, hemocompatibility, long-term durability, calcification, and transcatheter application. The latter portion of this review summarizes the currently available clinical outcomes data regarding polymeric heart valves and discusses future directions of research.

## Introduction

Valvular heart disease remains a prominent source of cardiovascular morbidity and mortality. The American Heart Association estimates that 2% of the United States population suffers from valvular heart disease, most often calcific aortic stenosis (AS) (1). Progression of valvular stenosis, seen mostly in patients older than 65 years, can present with worsening angina, syncope, heart failure, and mortality if left untreated. The gold standard treatment is surgical valve replacement.

According to the latest study, there were over 180,000 heart valve replacements performed in the United States in 2020 (2).

The current standard of care with respect to valve replacement offers patients the choice between mechanical and bioprosthetic valves, however limitations exist with both therapies. Mechanical valves, usually made from pyrolytic carbon, offer excellent long-term durability however require life-long anticoagulation for the patient, which comes with increased risks of bleeding and stroke (3, 4). Bioprosthetic valves, constructed from porcine valves or processed bovine pericardial tissue, do not require long-term anticoagulation but suffer from late structural valve degeneration (SVD) necessitating repeat valve intervention usually after 10–15 years (5). More recently, transcatheter valve replacement (TAVR) has grown more common as a minimally invasive alternative to traditional open surgical valve replacement. While initially conceived for patients at high risk for open surgery, its use in lower risk patients has grown as data has shown comparable outcomes with surgical valve replacement (6, 7). Similar limitations of surgical bioprosthetic valves exist for TAVR, specifically with regard to long-term durability (8). Moreover, interactions between the TAVR stent frame and valve leaflets that occur during crimping and deployment may also accelerate SVD (9, 10).

Ultimately, the ideal heart valve replacement will possess several characteristics (a) optimal hydrodynamic performance (b) biocompatibility (c) low thrombogenicity (d) applicability for TAVR and (e) low cost and widespread accessibility. Flexible leaflet polymeric heart valves (PHV) represent an emerging technology that may offer a solution to many of the aforementioned limitations present with currently available therapies. Though flexible leaflet PHVs were first implanted in the 1960s, widespread adoption of this technology has been limited by biodegradation and subsequent mechanical failure demonstrated through *in vivo* studies (11, 12). However, newer biocompatible and biostable polymers have shown great promise and have led to a resurgence in interest in this field. Moreover, PHV fabrication techniques allow for automated manufacturing leading to high reproducibility and lower costs.

**Figure 1:**
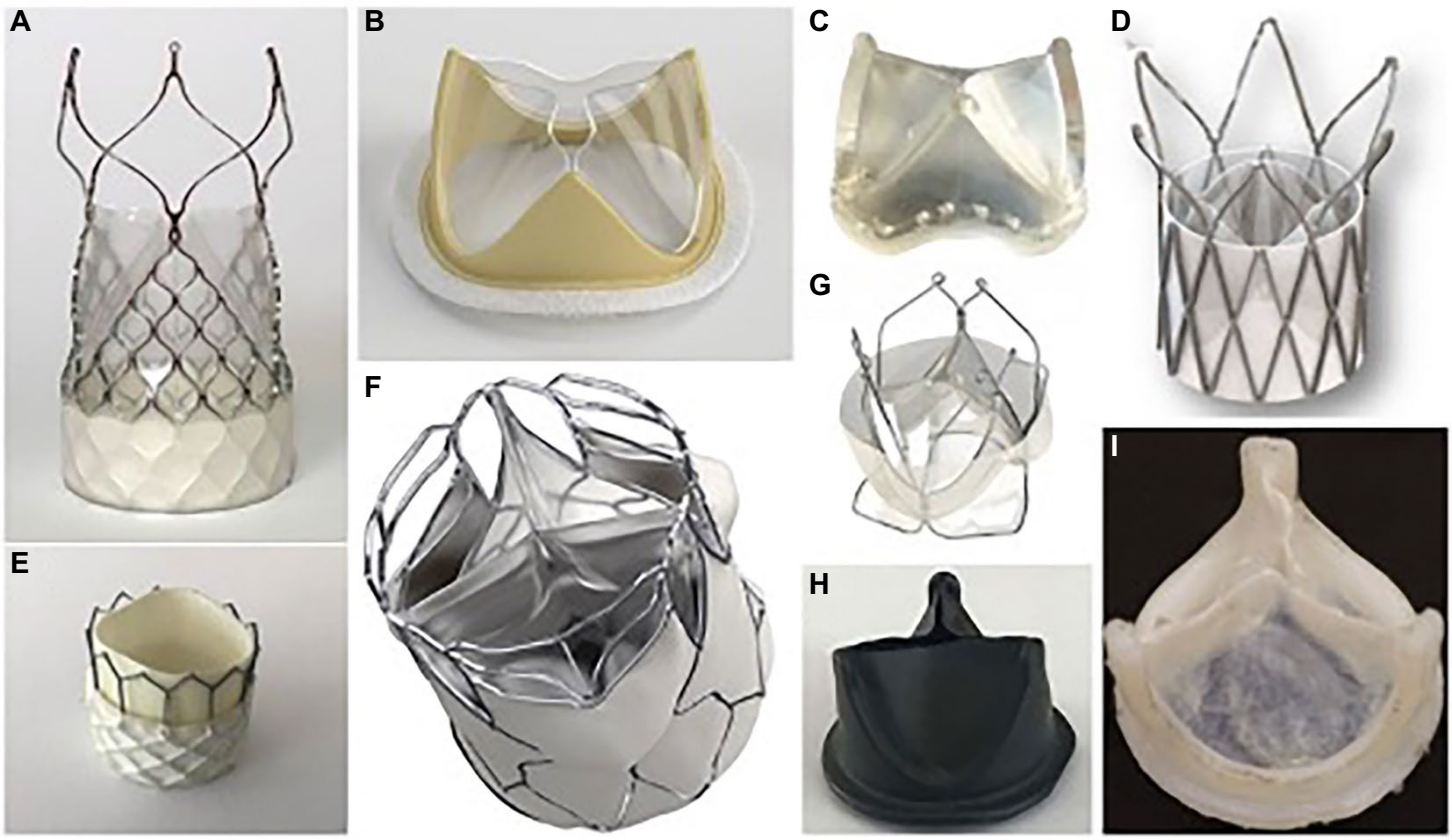
Selected polymeric heart prostheses. **(A)** Foldax Tria TAVR; **(B)** Foldax Tria SAVR A-M; **(C)** PoliValve; **(D)** Polynova TAVR; **(E)** Inflow TAVR; **(F)** SAT TAVR; **(G)** Triskele TAVR; **(H)** Hastalex; **(I)** Innovia.

In recent years, several flexible leaflet PHV options have been developed that are in various stages of clinical testing (Figure 1). Each valve has unique biomechanical properties that allow for optimized performance and durability (Table 1). The aim of this review was to summarize the current landscape of emerging PHV technologies and discuss the characteristics required for development of a successful valve replacement therapy.

**Table 1:** Description of various polymer valves, leaflet material, unique structural leaflets, and current status of development.

Device name	Leaflets material	Type	Features	Development status
Foldax Tria (Foldax Inc.)	SiPUU (LifePolymer [LP])	TAVR SE	⅓ thickness of bio tissue, no need for lifetime anticoagulation; NiTinol frame, 19F OD delivery system	Currently in preclinical testing—completed pilot study in sheep model. Foldax plans to conduct a larger pre-clinical study in 2022 intended to support a first-in-human study.
Foldax Tria (Foldax Inc.)	SiPUU (LifePolymer [LP])	SAVR A-M	Flexible LP leaflets solution-cast onto a radiovisible polyether-ether ketone stent with a PTFE felt sewing ring	Ongoing first-in-human early feasibility study in United States—1-year results published. Clinical trial in India started enrolling in May 2022.
Inflow (I4HV Inc.)	Copolymers of PU-PUS	TAVR BE	Cobalt-chrome alloy frame and a tri-leaflet polymeric valve connected with cuff; 15-16F OD	Completed initial animal trial in sheep model. Expected to conduct large GLP animal trial intended to support a first-in-human study.
Polynova (PolyNova Cardiovascular Inc.)	xSIBS polymer	SAVR	NiTinol frame, 16F OD, variable leaflet thickness profile and a semi-open nominal shape	*In-vitro* testing
SAT (Strait Access Technologies Inc.)	Triblock polyurethane combining siloxane and carbonate segments (Carbosil)	TAVR BE	Nickel-cobalt-chromium frame, self-elevating inter-commissural anchoring arms, also designed for AR	*In-vitro* testing
Triskele (UCL Cardiovascular Engineering Laboratory)	Urethane (POSS-PCU) polymer	TAVR SE	NiTinol frame, fully retrievable after deployment	Completed animal trial in sheep model. Enrolling pts. to first-in-human study in Q1 2023.
PoliValve (Universities of Cambridge and Bristol)	SEBS copolymer	SAVR	Made entirely of polymers (both leaflets and supporting structure)	Tested *in-vitro* and in short term *in-vivo* study.
Hastalex (NanoRegMed Inc.)	Functionalized graphene oxide and poly(carbonate-urea) urethane	SAVR prototype	Made entirely of polymers	Tested *in-vitro* and in short-term biocompatibility/calcification *in-vivo* studies.
Innovia (Innovia LLC.)	xSIBS	SAVR	Made entirely of polymers	*In-vitro* testing

TAVR, transcatheter aortic valve replacement; SAVR, surgical aortic valve replacement; SE, self-expanding; BE, balloon expandable; OD, outer diameter.

## Polymer compounds and materials

**Figure 2:**
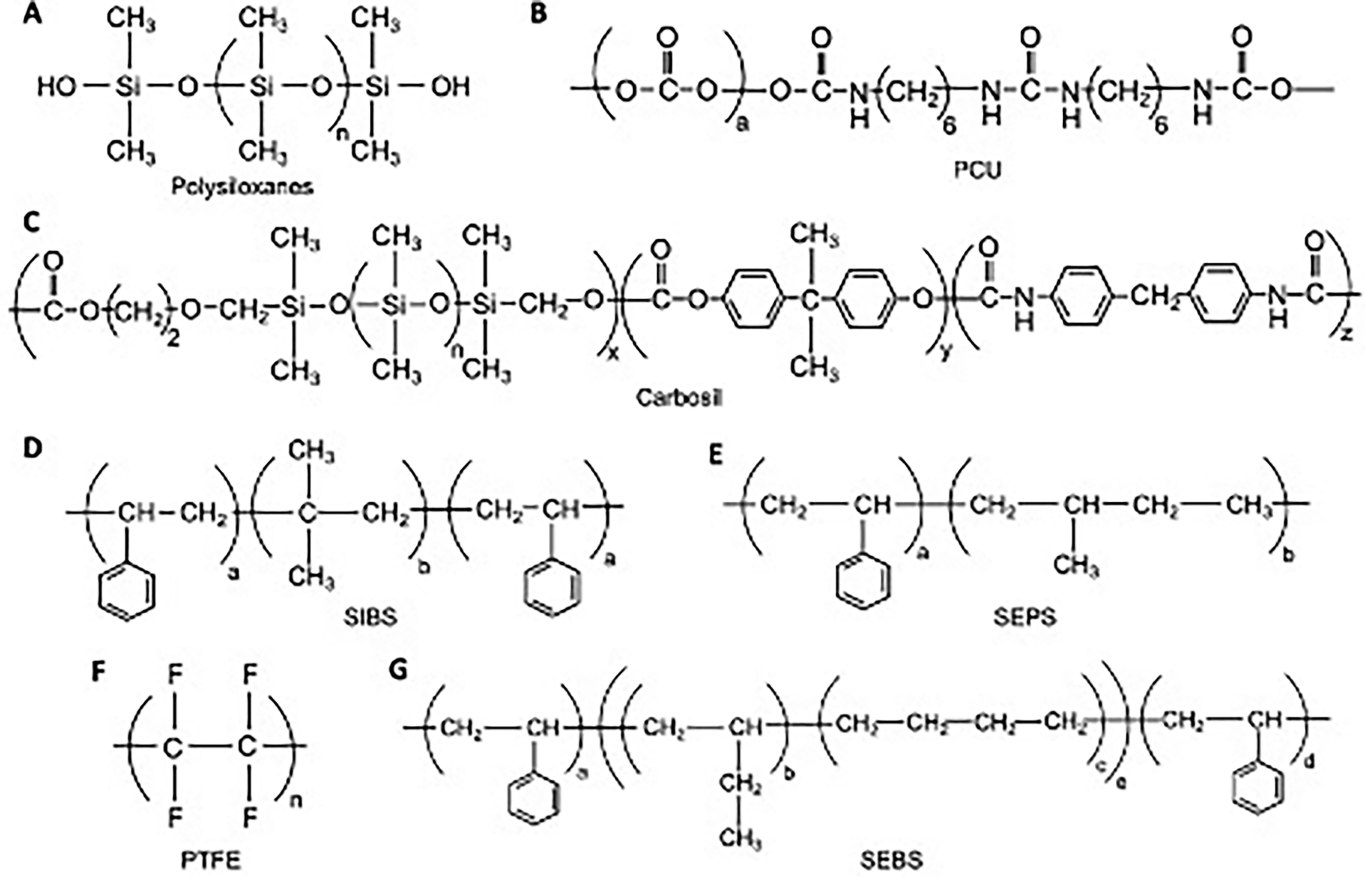
Chemical structures for various polymer compounds used in PHV construction. **(A)** Polysiloxanes; **(B)** Polycarbonate urethane; **(C)** Carbosil TSiPCU; **(D)** Poly(styrene-b-isobutylene-b-styrene); **(E)** Poly(styrene-b-isobutylene-b-styrene); **(F)** Polytetrafluoroethyelene; **(G)** poly(styrene-b-ethylene/butylene-b-styrene).

Unique to PHVs is the ability to tailor raw polymer materials to more closely match the properties of native tissue. The primary properties sought after during material design are (1) resistance to the highly dynamic loading conditions of the heart, (2) biostability to reduce the need for anticoagulation and (3) biocompatibility to minimize immune response, foreign body reaction, and long-term SVD. Additional properties may be pursued during material design, including growth capacity, stimulation of native tissue regeneration or stimulation of endothelialization. An array of polymer compounds exist that are both commercially available and in investigational use (Figure 2). Here we describe the most common polymers being used for the construction of PHVs and their respective properties.

### Polysiloxanes

Polysiloxanes (Figure 2A), or silicones, have been used in various medical devices such as gels, adhesives, vessels, and heart valves due to their excellent biostability, biocompatibility, and fatigue resistance. Much of the early work in the development of PHVs, mainly in the 1950s and 1960s, utilized silicone-based polymer compounds (13, 14). These early studies demonstrated acceptable early function *in vitro*, however long-term durability suffered *in vivo*, with reports of leaflet damage and embolic phenomena in large animal trials. Due to these concerns, silicones have largely not been used in experimental PHV development since the 1980s.

### Polytetrafluoroethyelene

Polytetrafluoroethyelene (PTFE), is a unique polymer (Figure 2F) known for its inertness and low surface energy, which has led to its use in various medical devices such as stents, vascular grafts, and defect repair membranes. Braunwald and Morrow first reported the use of a PTFE based valve implant in a clinical trial of 23 patients (15). Expanded PTFE (ePTFE), commonly known as Gore-Tex, was first used as valve material in a sheep tricuspid model (16). In both these studies, valve leaflets tended to stiffen and calcify. Subsequently, PTFE based polymers have not been widely used for valve manufacturing in light of these limitations.

### Polyurethanes

Polyurethanes represent a broad class of polymer compounds with a diverse array of architectures, which has led to many medical applications and specifically with many of the most recent PHV developments. Polyurethane-based leaflets contain hard and soft segments which allow for flexible leaflet motion. One major concern has been that these polymers contain degradation-susceptible bonds that may limit durability when exposed to oxidative conditions, metallic ions, enzymes, or mechanical stress (17). However, many improvements have been made since its initial incorporation in the 1950s that have allowed for more durable arrangements.

Polycarbonate urethane (PCU) is a modified polyurethane (Figure 2B) that has shown promise. ADIAM life science, a company out of Germany, developed aortic and mitral prosthetic valve using PCU (18). *In vitro* testing of the mitral prosthesis demonstrated up to 20 years of durability while *in vivo* testing showed relative resistance from SVD compared to bioprosthetic implants.

PCU has also been further modified with the addition of novel nanocomposite materials. One example of this is the inclusion of a nanocomposite of polyhedral oligomeric silsesquioxanes (POSS-PCU). The addition of the POSS group has been shown to protect the soft segment of PCU from oxidative and hydrolytic damage (19). Studies have also shown greater thromboresistance compared to PTFE (20). The POSS-PCU material has been used for vascular grafts and demonstrated good biostability and biocompatibility. More recently, it has been incorporated into the Triskele TAVR valve (21). Another example of nanocomposite modification is found in Hastalex material. With this technology, Ovcharenko et al. (22) has integrated functionalized graphene oxide (FGO) nanomaterials into a backbone of PCU. This material has already found application in recreation of tendons, urethra, and abdominal membranes, and is now being incorporated into a modern PHV model.

Siloxane based polyurethane materials have also been used for PHV development. The SAT (Strait Access Technologies, South Africa) TAVR valve incorporates a PCU-based polymer, Carbosil 2080A TSiPCU (Figure 2C), which combines siloxane segments for biostability and carbonate segments for strength into a polyurethane base (23). Recently, a siloxane polyurethane-urea (LifePolymer, LP) was specifically developed for heart valve leaflets (24). This compound has shown satisfactory biomechanical function and has led to the development of the Tria Surgical Valve (Foldax, United States). The copolymers polyurethane-co-carbonate (PU) and polycarbonate-co-silicone (PUS) have been used to prepare valve leaflets for the Inflow Artificial Transcatheter Heart Valve (ATHV; Cardflow consortium).

### Styrenic polymers

Styrenic triblock copolymers (STCPs) are synthetic thermoplastic elastomers that have applications in various medical devices, such as drug-eluting coronary stents. A novel PHV, PoliValve (University of Cambridge and Bristol, United Kingdom), has incorporated two STCPs, poly(styrene-b-ethylene/propylene-b-styrene; SEPS) and poly(styrene-b-ethylene/butylene-b-styrene; SEBS) (25) (Figures 2E,G). The unique ability of these compounds to self-assemble into a cylindrical microstructure allows for the production of structural anisotropy, with different mechanical properties in two orthogonal directions. Not only does this mimic native heart valve tissue, but also may enhance durability (25).

Poly(styrene-b-isobutylene-b-styrene), known as SIBS (Figure 2D), is a unique compound developed by Innovia (Innovia LLC, United States). The Innovia valve leaflets also incorporate a reinforcing Dacron mesh, however early attempts at surgically implanting this valve led to exposure of the reinforcing mesh and resultant calcification and thrombosis (26). A more recent polymer design xSIBS, is a cross-linked version of SIBS, and has shown promising *in vitro* results for TAVR application in the Polynova valve along with excellent hemocompatibility and resistance to calcific deposition (27, 28).

## Hydrodynamic performance

Prosthetic valve hydrodynamic performance is assessed in several different ways. The mainstay form of assessment is *in vitro* testing in a pulse duplicator system, where effective orifice area (EOA), mean pressure gradient (MPG) and regurgitant fraction can be quantified under a standard set of hemodynamic conditions. This allows a comparison of performance across valves. ISO 5840-3 has set minimum hydrodynamic function requirements for prosthetic valves (EOA ≥ 0.85 cm^2^, transvalvular regurgitant fraction ≤ 10%, total regurgitant fraction ≤ 20%). Here we summarize data that has been published regarding hydrodynamic performance of several PHVs that are currently being investigated. The following valves are discussed below: Triskele (POSS-PCU), SAT (TSiPCU), Foldax Tria (SiPUU), Polynova (xSIBS), PoliValve (SEPS/SEBS).

### Triskele (POSS-PCU)

The Triskele PHV TAVR valve (University College London Cardiovascular Engineering Laboratory, United Kingdom) *in vitro* hydrodynamic assessments have been previously reported (21). In this study, performance was compared directly with two commercially available TAVR devices (Edwards SAPIEN XT and Medtronic CoreValve). Hydrodynamic parameters were collected in various size aortic root models (21, 23, 25, and 27 mm) and 23, 26, and 29 mm valve sizes were used.

The authors report generally comparable hemodynamic parameters for the Triskele valve compared to both commercial TAVR devices during systole. However, in the 21 mm aortic root model, the Triskele valve demonstrated higher MPGs and lower EOA than controls. The Triskele valve demonstrated superior freedom from paravalvular leakage and total regurgitation in all aortic root sizes (21). The authors attribute this finding to the unique sealing cuff of the Triskele valve (Figure 1G). This cuff, also made from the same polymer material as the leaflets, surrounds the entire valve and covers the gaps between the prosthetic and native tissue. It remains to be seen how this feature may affect coronary re-access.

### SAT valve (TSiPCU)

The SAT polymer TAVR valve’s hydrodynamic performance was tested *in vitro* (29). All parameters met minimum criteria set by ISO standards. In this study, the polymeric TAVR valve was compared to a bioprosthetic SAVR (Edwards Perimount) and TAVR valves (SAT bioprosthetic TAVR). Interestingly, the polymer valve had significantly thinner leaflets compared to bioprosthetic counterparts (152 um vs. 602 and 403um). The bioprosthetic TAVR valve had a lower pressure gradient and higher EOA than the polymeric TAVR valve and the bioprosthetic SAVR. All valves exhibited similarly acceptable, low regurgitant fractions.

### Foldax Tria (SiPUU)

The Foldax Tria surgical aortic valve (Figure 1B) has reported similar EOA and pressure gradients compared to Edward Perimount valves during pulse duplicator hydrodynamic testing (30). More specifically, under normotensive hydrodynamic testing *in-vitro*, a MPG of 7.3 mmHg, EOA of 2.4cm^2^, and regurgitant fraction of 3.9% have been reported.

### Polynova (xSIBS)

**Figure 3:**
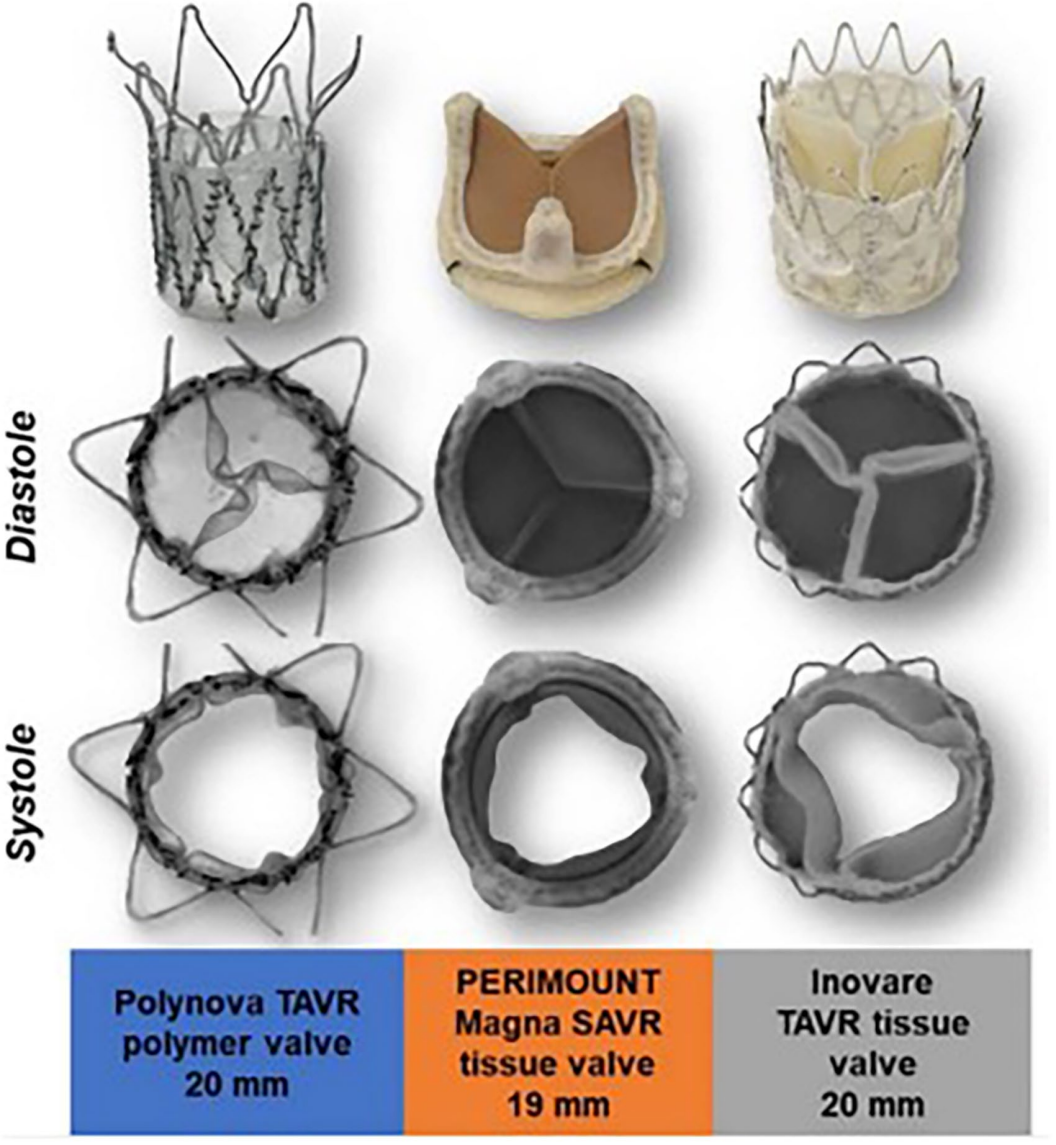
Polynova polymer TAVR valve compared with Perimount Magna SAVR and Inovare TAVR tissue valves. Images from *in-vitro* testing showing valve in peak diastole and systole.

The Polynova TAVR valve underwent *in vitro* testing for hydrodynamic performance and was compared to standard bioprosthetic SAVR (Perimount Magna Ease) and TAVR (Innovare) valves that are currently in clinical use (27). The authors found that during hydrodynamic testing, the Polynova PHV had a larger EOA compared to other valves (Figure 3). The Polynova valve also had a lower MPG than the TAVR valve, but a higher MPG than the SAVR valve. Additionally, the Polynova valve had a higher regurgitation fraction (11.87%–21.28%) than both the SAVR (1.97%–2.48%) and TAVR (7.26%–9.39%) bioprosthetic valves.

One explanation proposed by the authors for the higher regurgitant fractions seen in the polymeric TAVR valve is that the addition of a sleeve in the leaflet region for stent attachment may reduce the size of the neo-sinuses which prevents the formation of a strong recirculation zone in the sinuses, leading to delayed closure of the leaflets and increased regurgitation (27) (Figure 1D). This design feature is reportedly being addressed in future models of this valve.

### PoliValve (SEPS/SEBS)

The PoliValve (Figure 1C) underwent has undergone similar hydrodynamic testing. In this study, authors manufactured identical polymer valves but varied leaflet thickness from 0.24 to 0.46 mm (25). All valves met minimum ISO standards for hydrodynamic performance, however they found significant differences in hydrodynamic parameters based on leaflet thickness. EOA showed a marked reduction (from 2.5 to 1.1 cm^2^) with increasing leaflet thickness. MPG also increased (from 8 to 25 mmHg) as leaflet thickness increased. With regard to regurgitant fraction, this generally decreased with increasing leaflet thickness. When compared to the Edwards Perimount valve, hydrodynamic parameters of the PoliValve with leaflet thickness of 0.35–0.40 mm were generally similar (25).

## Long-term durability

Durability of prosthetic valves is tested using an Accelerated Wear Testing (AWT) model. This model focuses into durability in response to repeated mechanical performance and pressure and does not take into account host factors affecting the material. The WST model includes a pump that enables repeated opening and closing of the heart valve prosthesis under accelerated conditions. Test conditions are specified by ISO 5840, which requires a 100 mmHg pressure difference across the closed aortic valves for at least 5% of each cycle, maintained for more than 95% of all test cycles. The minimal number of cycles to complete is 200 million, an equivalent of 5 years of normal function. Devices are inspected (microscope, photographs, and high-speed video) and functionally evaluated under pulsatile flow at least every 50 million cycles. Here we present the available reports concerning durability of several polymeric valves.

### Triskele (POSS-PCU)

Manufacturer briefly reports that the developed device met and largely exceeded the durability requirement for flexible leaflets prosthetic valves (>200 million cycles) (21), however detailed testing procedures are not available.

### SAT (TSiPCU)

The polymeric valve was tested in AWT at 15 Hz and reached the predetermined 400 million cycles. No signs of macro-degradation such as delamination of the free leaflet edge were seen as reported by the authors (23).

### Foldax Tria (SiPUU)

The Foldax Tria valve has reported that in AWT they have observed minimal structural damage up to 670 million cycles (equivalent of 16 years) (30). Their durability testing is ongoing as of the latest reports in 2020.

### PolyNova (xSIBS)

To assess the baseline durability of the PolyNova valve design itself, the polymeric valves were tested without the stent frame (31). The durability testing included four valves mounted in AWT with a 3D-printed rigid sleeve as a support, excluding the effects of crimping, and oversized deployment. The authors report that all prostheses surpassed 400 million cycles (up to 900 M) (27). They noticed no visual damage to the leaflets, as well as no changes to the effective orifice area that was kept at an average of 1.8 ± 0.04 cm^2^. Additionally, they observed that pressure gradients were stable up to 200 million cycles (14.6 ± 0.8 mmHg), with a gradual decline afterwards (10.4 ± 0.9 mmHg). Interestingly, the closing regurgitation declined linearly within the first 350 million cycles with a slight peak reported at 400 million (10.1% ± 0.9% to 6.5% ± 0.8% and 7.5% ± 1.0% respectively).

### PoliValve (SEPS/SEBS)

For durability testing, several different prototype materials were compared. The testing method was similar to the ones described above, utilizing AWT (25). Valve failure was identified by abnormal proximal and distal pressure traces, followed by visual inspection. Authors reported marked improvement of successive prototypes from 13 million cycles to >1.2 billion (*p* > 0.0001).

**Figure 4:**
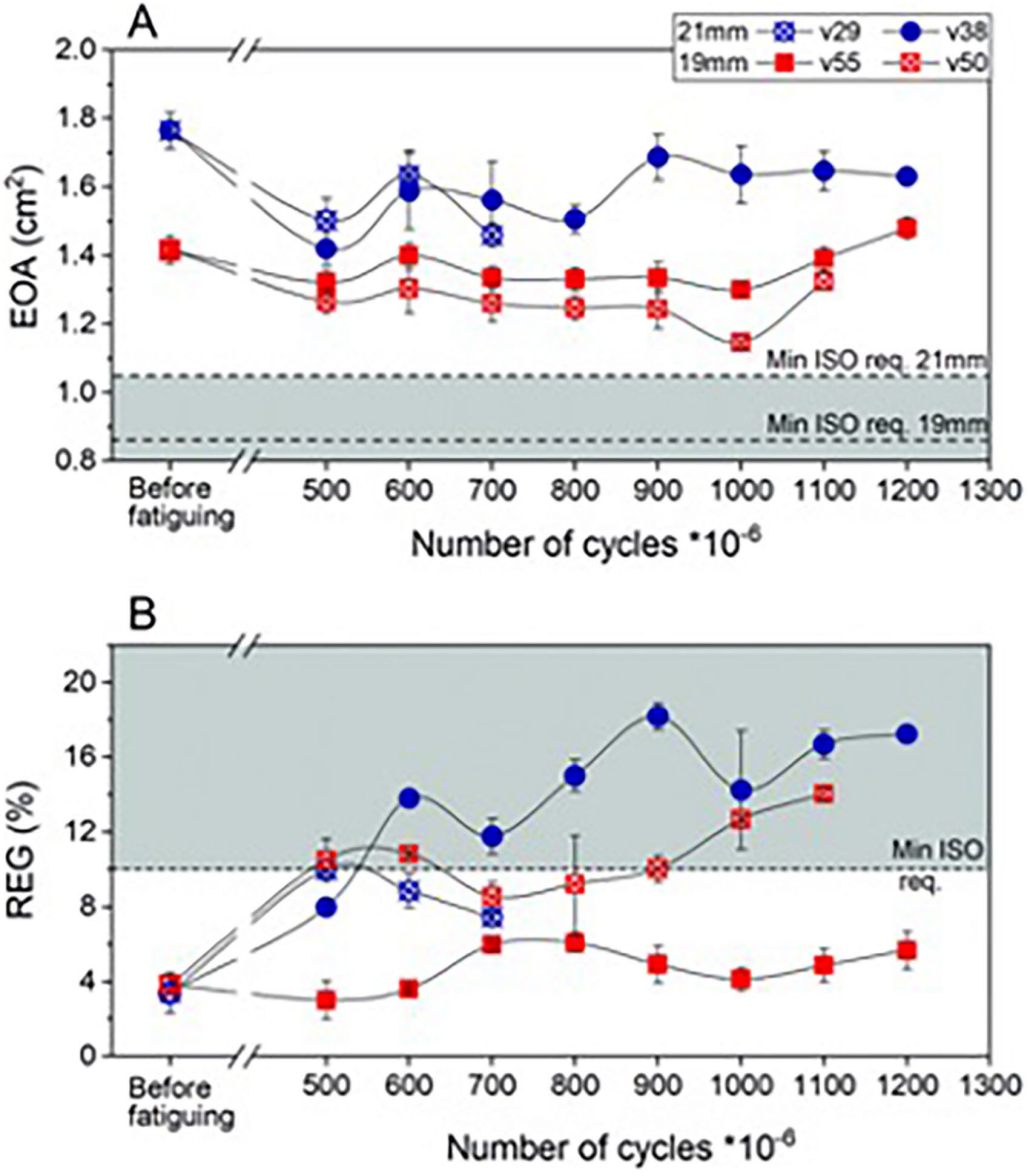
Hydrodynamic performance of fatigued PoliValve prototypes of various iterations. **(A)** Effective orifice area (EOA); **(B)** regurgitant fraction (REG). Durability testing performed over 1.3 billion cycles.

All four latest prototype valves (21 mm and 19 mm, two each) greatly surpassed the required 200 million cycles. Hydrodynamic performance of each valve was measured before fatiguing and repeated after 500 million and then every 100 million cycles (Figure 4). One device failed after 783 million due to a vertical crack near the commissure. The researchers highlight that the leaflets of this valve also showed some minor defects at the free edge. The free edge of two remaining valves also started to show some damage beyond 500 million cycles. For the three remaining valves, 1.1 billion (1 valve) and 1.2 billion cycles (2 valves) were passed, respectively. Nevertheless, growing fatigue influenced the effective orifice area (EOA) as well as regurgitation intensity. Researchers report that beyond 500 million cycles the opening area decreased by 10%–15% on average as compared to baseline. However, EOA of the valves remained well above the minimum required by ISO standards (0.85 cm^2^ for 19 mm and 1.05 cm^2^ for 21 mm). Only one 19 mm prosthesis remained fully functional at 1.2 billion cycles (EOA = 1.48 cm^2^, REG = 5.66%), an equivalent of over 30 years of operation. For the remaining 2 valves, the regurgitation fraction exceeded 10% at 600 million (21 mm) and 1.1 billion (19 mm) cycles, respectively. This was still smaller than the maximum of 20% as allowed by ISO. The authors concluded that in general the 19 mm valves performed better than the larger counterparts. They attributed this result to slightly thicker leaflets of the 19 mm prostheses, which is a result of minor mold variability.

### Inflow ATHV (PU-PUS)

The authors report that six artificial heart valves were tested using BDC Laboratories VDT-3600i pump. Waveform, frequency, and stroke conditions were all adjusted in the same manner as all tested prototypes. All valves passed 40 million cycles and further tests are ongoing (32).

## Thrombogenicity and hemocompatibility

PHVs hold a promise of significantly reducing the thrombotic risk compared to mechanical prostheses, and thus consequently limiting the amount of anticoagulation required. Despite the encouraging results of various preclinical studies, no PHV with a well-established anticoagulation protocol exists. The ultimate potential of a polymeric valve for widespread applicability will rest on the ability to discontinue systemic anticoagulation long-term. Similar to bioprosthetic tissue valve, it is reasonable to expect a period of short-term anticoagulation until a non-thrombotic environment has been created.

Thrombogenicity and hemocompatibility of PHVs have been evaluated using a variety of *in vitro* and *in vivo* models that aim to mimic human-like conditions and thoroughly investigate blood-implant interactions. ISO 10993:4 describes in detail the requirements for testing. Considering the novelty of investigated devices/materials, tissue valves and clinically used materials (such as Dacron grafts) are often used as a comparator. Below we report data that has been published regarding thrombogenicity of five PHVs currently being investigated.

### Hastalex (FGO-PCU)

To assess hemocompatibility, the Hastalex (Figure 1H) and GORE-TEX (control) samples were incubated in a citrated human blood/saline mixture (22). The absorbance of supernatant was measured on a spectrophotometer. Additionally, to determine the maximum aggregation of blood platelets, samples were incubated with platelet-rich plasma. Authors report that there was no negative effect on red blood cell membranes for both types of test materials. No statistically significant differences were noted in the degree of hemolysis between Hastalex and GORE-TEX samples. Similarly, the maximum platelet aggregation was comparable between the tested materials (*p* = 0.62). Interestingly, the degree of platelet deformation on GORE-TEX was higher than that of the Hastalex surface. The platelet strain index of GORE-TEX was 2-fold higher than that of Hastalex (*p* = 0.03) (22).

### Foldax Tria (SiPUU)

The *ex vivo* thrombogenicity test for the Tria valve utilized a nonhuman primate (NHP) AV shunt method (33). Six LifePolymer (LP) and 5 ePTFE grafts were compared (24). LP grafts confirmed good hemocompatibility with minimal platelets deposition [<0.08 × 10 (9)] for the entire 60-min study period. ePTFE grafts underwent significantly (*p* < 0.05 in *t*-test) higher platelet attachment at 30, 45, and 60 min. The LP result was an order of magnitude below historical results on ePTFE grafts with dual antiplatelet therapy, which had 2.0 ± 0.4 × 109 platelets after 60 min. The average amount of fibrin present on the LP devices was significantly lower than on the control (0.003 ± 0.007 mg vs. 0.844 ± 1.156 mg; *p* = 0.027). Additionally, 8 surgical Tria valves (23 mm) and 2 Carpentier-Edwards Perimount aortic valves (25 mm; control) were implanted in a sheep model for a 140-day period. Histopathology reported no surface thrombus on either LP or control valves (24).

The Foldax Tria surgical valve is the only PHV that has been tested in humans (14 patients; 1 year follow-up) (34). According to the study, anticoagulation with warfarin was initiated postoperatively, with a target international normalized ratio of 2.0 to 2.5 and was continued for 6 weeks with transition to aspirin 75 to 100 mg/d as tolerated. Two patients died due to valve unrelated causes (bleeding from elective surgery; hemodynamic collapse and cardiac arrest due to possible pulmonary embolus after warfarin discontinuation in a morbidly obese subject—normal valve function). One patient died due to the lacunar stroke on day 172. Additionally, an acute myocardial infarction (day 92) from thrombotic obstruction of the right coronary artery was observed. Computed tomography confirmed a thrombus possibly involving the valve sewing ring. The authors report that the above was successfully treated with stent implantation followed by 6 months of dual antiplatelet therapy plus warfarin (34).

### Innovia (SIBS)

The Innovia PHV (Figure 1I) was compared against Carpentier Edwards Perimount Magna tissue valve in a platelet aggregation test (35). Using inflow/outflow valve holders to control unidirectional flow, a pair of 19 mm valves (test vs. control) was mounted into a Berlin pulsatile left ventricular assist device (LVAD) circuit. An LVAD running without prostheses constituted a negative control. The flow loop system was filled with modified Tyrode’s platelet buffer solution and human gel filtered platelets. Flow cytometry and prothrombinase platelet activity state (PAS) assays methods were used. Platelet activation rates (PAR) were, respectively, fivefold (*p* = 0.005; PAS) and fourfold (*p* = 0.007; flow cytometry) lower in the Innovia valve than in a tissue valve (35). Negative control tests (*n* = 6) conducted with the LVAD without the valves *in situ* had significantly lower PAR value.

### Polynova (xSIBS)

The Polynova TAVR valve was compared against Carpentier-Edwards Perimount Magna Ease tissue prostheses and Innovare TAVR valves using a closed flow-loop mock silicone left ventricle model (27). The circuit was filled with a human gel-filtered platelet buffer and a pulsatile reciprocating pump was used to compress/relax the ventricle model and generate flow. Each test was run for a duration of 30 min. The polymeric valve demonstrated the least thrombogenic potential, followed by the SAVR valve (with non-significant difference), and lastly the Innovare valve (*p* < 0.005) with an increase in the platelet activation rate by a factor of 6.5 as compared to the polymer valve (27).

### PoliValve (SEPS/SEBS)

Thrombus formation was assessed using the Badimon chamber that consists of a pump and three perfusion chambers that simulate different rheological conditions (25). Strips of material (both heparin-coated and uncoated), cut to fit inside the chambers filled with human blood, acted as the thrombogenic substrate. Each study lasted for 5 min, during which flow was maintained at a constant rate of 10 mL min^−1^. All samples demonstrated good hemocompatibility, regardless of coating. Contrary to what is usually seen with porcine aorta as a substrate, no thrombus aggregation was reported on the polymer strips. Furthermore, previous studies have shown that heparin coating of similar polymers reduces surface thrombogenicity. Its stability, as established in *in vitro* models, was proved to exceed 500 h (36). Tested valves retained 60% of its surface coating after 190 million cycles (4.5 years equivalent) in the accelerated fatigue tester (36).

### Inflow ATHV (PU-PUS)

The polymeric valve was tested in a chronic large animal model (32). Sixteen sheep underwent the TAVR procedure. After a designated observation period the animals were euthanized, and valves were subjected to histopathology analysis. Two animals died prematurely due to cardiac complications secondary to valvular regurgitation (infection and vegetation on the prosthesis). As indicated, three animals were sacrificed after 30, four after 90, and six after 180-day follow up. Authors report that both at 30 and 180 days the leaflets were completely free of thrombi. Only between the base of the leaflets and aortic wall a thin layer of clots was formed (30 days group). However, at 90 days in three animals a thin thrombus was visible on the ventricular surface, presenting as surface deposits, well delineated and firmly attached to the base of the leaflets and adjacent stent elements contacting the aortic wall. Overall, the changes reported did not influence the valve hemodynamics as reported by the authors (32).

### Triskele (POSS-PCU)

The hemocompatibility of the nanocomposite POSS-PCU was previously compared with PTFE material (37). The samples were exposed to the citrated human blood (and extracted platelet rich plasma). To assess coagulation, pieces of material were placed in cups that were filled with blood and mounted onto the TEG 5000 Thromboelastograph Hemostasis System. Platelet adhesion test was performed by placing samples in separate tissue culture plates and exposing them to platelet rich plasma. The degree of platelet adhesion was measured *via* the platelet adhesion index. Similarly, the whole blood clotting response was analyzed by filling the plates containing samples with citrated blood. Authors report that POSS-PCU showed lower thrombogenicity and higher hemocompatibility comparing with porous PTFE on the aspects of platelet activation, adhesion and whole blood reaction (37).

## Immune response and calcification

Calcific degradation constitutes yet another major challenge for valve manufacturers. Growing calcific masses can immobilize leaflets leading to structural valve deterioration. The process itself can be either passive where calcium ions accumulate disrupting the tissue or active where the inflammatory responses to antigens present in prosthetic tissues leads to accelerated tissue mineralization (38, 39). αGal, a carbohydrate expressed in animal-derived tissue cells, and anti-αGal antibodies are noted to result in hyperacute xenotransplant rejection in nonhuman primates (40). Bovine and porcine bioprosthetic valves are coated with a rich set of glycosphingolipids and glycoproteins, including Neu5Gc and αGal (41). Recent studies have shown that bioprosthetic tissues engineered to be deficient in Neu5Gc and αGal could be less likely to undergo immune-mediated deterioration (42, 43). PHVs have a unique advantage over traditional bioprosthetic materials in that they lack animal derived proteins such as Neu5Gc and αGal, which may result in longer durability.

While all the PHV options currently undergoing clinical testing are biocompatible (ISO 10993-6:2016) and have been tested for foreign body response and inflammatory cell recruitment, short-term and long-term immune responses are expected with PHV and may influence calcification and long-term durability. Proper selection of polymeric material for leaflets might help overcome calcific degradation, by engineering their chemistry to tackle both the passive and active calcific deposition. However, this requires thorough evaluation. Calcific susceptibility can be assessed using both *in vitro* (44, 45) and *in vivo* testing (46). A frequent method employs the use of chronic preclinical models. Sheep are the preferred choice given their accelerated and enhanced calcium metabolism that creates a “worst-case-scenario” in terms of valvular calcification. Processes to which heart valves are subjected within several months in juvenile sheep take several years to develop in human patients (38, 46).

### Hastalex (FGO-PCU)

Hastalex polymer material along with GORE-TEX (control) and GA-pericardium material were implanted subcutaneously in 5 Wistar young rats and left for 14 days (for foreign body response) or 60 days (for calcification) (22). Subsequently, the samples were harvested, histopathologic examination was performed, and the total calcium concentration was quantified by inductively coupled plasma optical emission spectrometry. Reported results showed that at 60 days, the fibrous capsule around Hastalex was thinner than that around GORE-TEX, suggesting advanced biocompatibility of Hastalex. Hastalex also had none to minimal calcium deposits contrary to the control materials that presented with higher calcium concentration (*p* < 0.05). The GORE-TEX samples in particular contained visible calcific deposits at the tissue/implant interaction.

### SAT (TSiPCU)

Decellularized and sandwich-crosslinked bovine and porcine pericardium as well as Carbosil (100% pre-strained on Co-Cr lattice frames to simulate the contact of the leaflets with valve stents) were compared with standard 0.7% glutaraldehyde fixed bovine pericardium (23). All samples were implanted into 5-week Long-Evans rats for 6 weeks. The authors report that the Carbosil material showed practically no calcification at all (0.28 ± 0.07 μg/mg), and significantly less as compared to even the decellularized tissue (*p* < 0.05) (23). The biocompatibility of Carbosil had been previously shown in cage implants (47). Strained materials were placed in 305 stainless-steel wire mesh cages and implanted subcutaneously in 3-month-old Sprague–Dawley rats. After an initial increase in leukocyte concentrations the mild inflammatory response resolved by day 14 post implant, with cell counts comparable between Carbosil and the empty mesh cages. Of note, presence of macrophages that fused into foreign body giant cells was noted after 21 days of implant in Carbosil. Foreign body giant cells were reported to be bigger and more abundant in Carbosil than in PCU without silicone modification. A more recent study that employed un-caged subcutaneous implants of Carbosil in 10-week Wistar rats did not find signs of calcification, macrophage infiltration, or foreign body giant cells after 60 or 90 days of implantation (48).

### Foldax Tria (SiPUU)

The Tria TAVR valves were delivered transfemorally and implanted within the aortic annulus that was previously prepared with a surgical annuloplasty ring (30). Authors report that nine sheep underwent the procedure, with 6 making it into a chronic study. Remaining animals were observed for 90 days, after which the valves were harvested and subjected to histopathology. Radiography as well as gross pathology showed no calcification or other adhesions/growth on the leaflet surface.

Additionally, the LifePolymer was assessed within a rabbit model (24). Samples were implanted subcutaneously above the dorsal muscle for 3–6 months. X-ray analysis found no evidence of calcium deposition on the material at either 3- or 6-month time points.

### PolyNova (xSIBS)

Calcification susceptibility was tested using a modified *in-vitro* protocol for accelerated testing (49). The valves were mounted in the AWT device. Similar conditions to durability testing were obtained except that the saline was replaced by Golomb and Wagner’s pro-calcific/phosphorus compound that resembles the ion concentrations of blood. The compound was replaced weekly and the valves were tested for 50 million cycles. Three polymeric valves (*n* = 3) were tested without the stent similar to the durability testing setup. 21-mm Carpentier-Edwards PERIMOUNT Magna Ease SAVR bioprosthetic valves (*n* = 2) were tested as a reference. Additional valve samples of both the PolyNova and the SAVR bioprosthetic valves (*n* = 1, each) that were not exposed to the AWT-calcification test were used as negative controls. After completion, the valve samples were removed and scanned in μCT and evaluated with mass spectroscopy. Authors report that μCT scan showed that mineralization of the tissue valves penetrated into the bulk of the leaflets. In contrast, no difference was seen in the polymeric valves between the test samples and the negative control. Spectroscopy confirmed that the normalized accumulation of both calcium and phosphorous in the polymeric valves was negligible, and significantly lower than the tissue valves by a factor of 85 and 16, respectively (*p* < 0.001) (49).

### Inflow ATHV (PU-PUS)

As described in previous sections, the Inflow TAVR valve was implanted in 16 sheep that were followed for 30 (*n* = 3), 90 (*n* = 4), or 180 (*n* = 6) days (32). Histopathologic examination as well as X-ray analysis showed no calcifications of leaflets, free margins, and commissures in all cases at 30 days. Inflammatory infiltrations were reported at 90 days, without cellular penetration into the polymer. At 90 and 180 days focal punctiform calcifications of leaflets and commissures were reported in 1 and 3 cases, respectively. The post-mortem analysis of two animals that died prematurely unveiled that in both cases a possible cause of death was heavy calcification of the banding region with subsequent infection and vegetation on the prosthesis that immobilized the valve. The authors argue that as seen in histopathology, the calcification process originated externally to the prosthesis (potential infection of the banding site during surgery) and then penetrated to the valve itself. As highlighted in the article, the above was confirmed by independent pathological analysis that qualified this event as banding model related.

### Triskele (POSS-PCU)

The POSS-PCU material calcification susceptibility was previously tested using a specially designed accelerated physiological pulsatile pressure system for a period of 31 days and a total of 4 × 10^7^ cycles (50). Samples of POSS-PCU, bovine pericardium (BP) and polyurethane (PU) were immersed in the physiological calcium solution (both calcium and phosphate in the same total concentration as those in human blood plasma). Subsequently, the samples were investigated for signs of calcification by means of X-ray, microscopic and chemical inspections. Comparison showed that, in the experimental conditions, the level of calcification for the nanocomposite was significantly lower than that for the fixed BP (*p* = 0.008) and PU samples (*p* = 0.015). Considerable calcium depositions were detected on the BP and PU samples, with only negligible reported on the nanocomposite surface.

## Clinical outcomes

**Figure 5:**
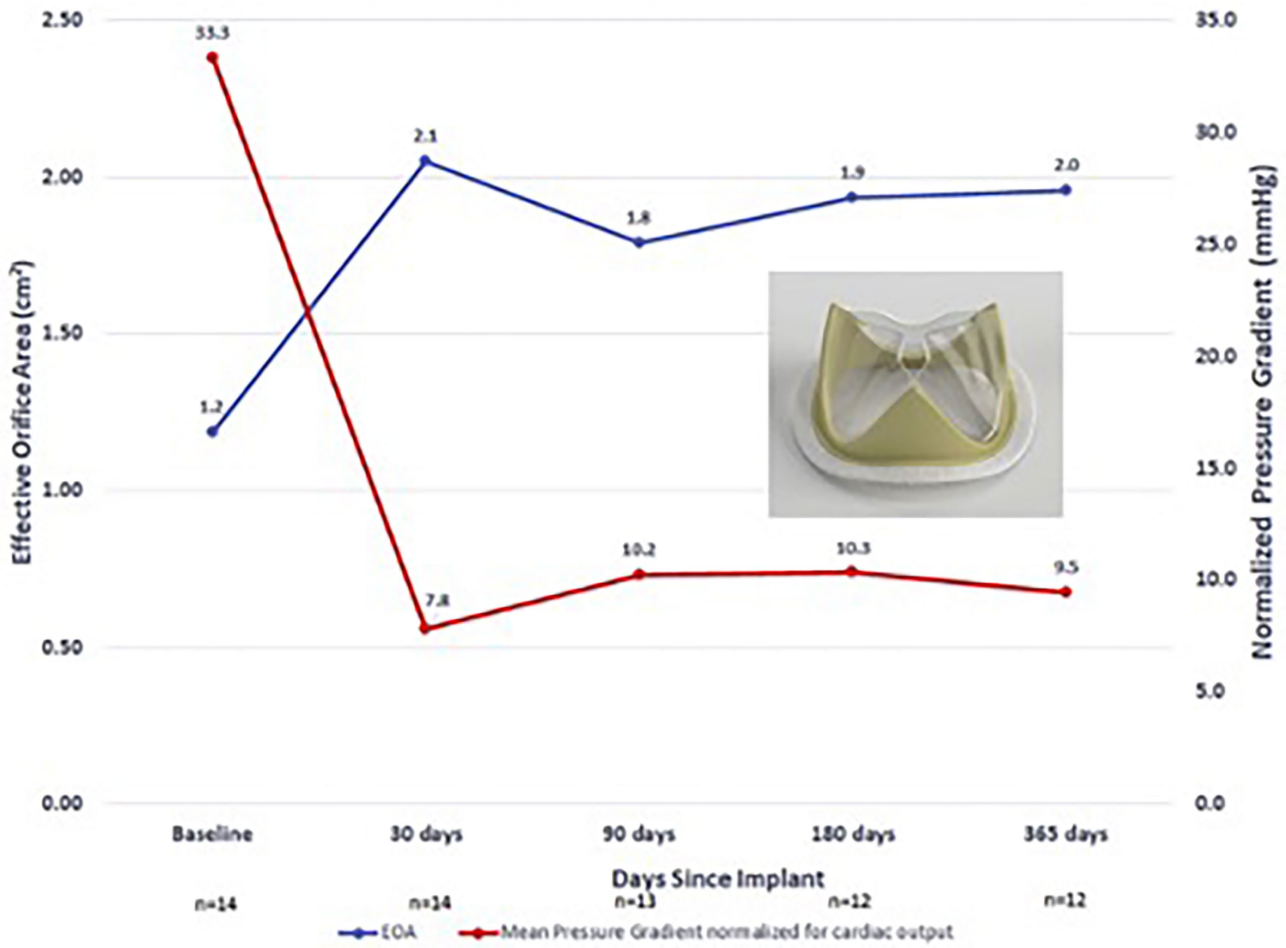
Foldax Tria surgical valve early feasibility study in humans—EOA and MPG over 1-year follow-up.

While several valves have been tested in large animal *in-vivo* models, clinical outcomes of polymer valve trials in humans are very limited. The only completed in-human trial that has been reported is the Foldax Tria surgical valve. The authors conducted a prospective, single-arm early feasibility study in patients with symptomatic aortic valve disease (34). Fourteen subjects were enrolled at 5 centers and subjects were followed up to 1 year following implantation. In terms of major adverse events, there were two deaths reported unrelated to the valve or procedure and one lacunar stroke 172 days following surgery. One patient also was reported to have myocardial infarction 92 days postoperatively from thrombotic obstruction of the right coronary artery possibly related to thrombus originating from the valve sewing ring. With regard to functional outcomes, New York Heart Association (NYHA) functional class was improved and sustained to 1 year across the cohort and MPG and EOA were improved postoperatively and maintained up to 1 year (Figure 5) (34).

## Endothelialization and PHV modification methods

A non-thrombotic environment can be achieved by endothelialization of the implant material (51). *In vivo* endothelialization of the PHVs has not been directly reported in the literature. The SAT valve, along with other PHVs, incorporates a degree of porosity in its electrospun skirt component (23). This porosity allows for capillary ingrowth across the thickness of the valve skirt, as seen in their bioprosthetic model (52). The authors suggest that this process of capillarization may allow for functional endothelialization in humans, though this remains to be demonstrated in *in-vivo* models. Coating or chemically binding polymeric materials with the extracellular matrix components is being explored in implantable material design, as an approach to reduce platelet adsorption (53) as well as to encourage *in vivo* endothelialization (54).

The risk of endocarditis associated with PHVs has not been directly explored. The risk of microbe attachment and formation of septic vegetations will likely depend on the degree of endothelialization that is able to be achieved on the PHV surface. Studies of other non-polymer cardiac grafts have demonstrated that aberrant endothelialization of graft material may explain the higher incidence of endocarditis seen with certain types of prosthetic materials (55). Further studies focused on PHVs will be needed to more clearly define the risks of endocarditis compared to bioprosthetic and mechanical alternatives.

Ongoing investigations in polymer materials aim to overcome additional challenges in PHV durability. Some of the emerging host mechanisms that impact durability of bioprosthetic materials may also affect PHV, such as oxidation or protein infiltration (56). Modification of Carbosil to reduce oxidative degradation increased durability in a preclinical model (48). Non-calcific degeneration due to proteinaceous infiltration has been reported in PTFE and may also impact newer materials (57). Proteinaceous infiltration after implantation or incorporating extracellular matrix components to PHV as a design approach to improve biocompatibility may make polymers more susceptible to chemical mechanisms known to affect bioprosthetic materials, such as protein glycation.

## TAVR application

Presented polymeric prosthetic valves hold a promise of addressing the current limitations of bioprosthetic SAVR/TAVR valves and thus create a viable alternative that will combine the durability of artificial material with low thrombogenicity. Out of the described devices, currently five have a particular focus on TAVR application. Polynova, Triskele and Foldax Tria (Figure 1A) are self-expanding valves, while Inflow (Figure 1E) and SAT TAVR (Figure 1F) valves are balloon expandable. The majority of these projects are still in early phases of preclinical trials with Polynova and SAT completing *in vitro* tests so far. Inflow and Foldax TAVR valves have already completed initial animal trials and are planning to conduct further ones to support first-in-human trials. Triskele has already completed initial animal trials and is scheduled to enroll patients for a FIH trial in Q1 2023. The detailed characteristics of each polymeric valve are presented in Table 1.

TAVR materials must be thinner than SAVR to accommodate for catheter delivery. Moreover, the polymeric TAVR valve manufacturers face an additional challenge in the form of crimping. Recently, a growing amount of evidence has shown that both valve crimping and subsequent expansion during deployment can cause irreversible mechanical damage to leaflet tissue (58–61). The extent is influenced both by the time and the desired crimping size (59, 62). The concern of crimping duration restricts most TAVR devices today to be crimped onto the delivery catheter and immediately deployed at the procedure site. Introduction of more durable materials such as polymers might have the potential to overcome this limitation and fuel a shift in paradigm (63).

Crimping stability and resistance to damage was extensively tested with the PolyNova TAVR valve (49). Stented polymeric valves were crimped to 16 Fr for 20 min or 8 days (3 valves per time point). The durations were chosen based on average crimping time during the procedure of commercially used bioprosthetic TAVR valves and for the longer period to replicate factory crimping with subsequent delivery to the operation suite. A 5 Fr catheter was placed concentrically within the valves to replicate crimping onto the delivery system. After completion, the crimping was slowly released, and the valves were self-expanded back to their nominal size. As reported by the authors, the visual inspection (using a 10× zoom camera) of the valves in both time points did not reveal any tear or damage to the valve neither in the sleeve nor in the polymeric leaflets. Light longitudinal folding marks were evident in the valves crimped for 8 days. These were macro-folding marks that were not evident in the SEM scans, nor were these associated with any plastic deformation or superficial damage on a micro-scale. The SEM scans focused in particular on three wide regions of the leaflets, which cover the connection regions of the leaflets, as well as the belly of the leaflets. These were chosen based on data of previously performed strain analyses (64–66). Importantly, this type of study has not been performed for the majority of PHVs described in this study.

## Conclusion

By the year 2050, it is estimated that over 1 million patients will require a heart valve replacement. The available prosthetic valve options that exist today have several limitations including lifelong anticoagulation for mechanical prostheses and structural valve degeneration associated with bioprosthetic valves. Moreover, the costs and materials required to source and process biological tissue limit the widespread utilization of bioprosthetic valves in low-resource settings. Early trials of polymeric heart valves were unsuccessful largely due to a limited ability to modify polymeric compounds to meet the needs of an ideal heart valve substitute. This review describes several advanced polymer technologies that have been incorporated into more recent PHV models.

In our assessment, PHVs represent an important prospect and have the potential to significantly alter the treatment of valvular heart disease. Studies of new PHV models have demonstrated satisfactory hydrodynamics and *in vitro* durability along with reduced thrombogenic and calcification potential in limited *in vivo* studies, compared to previous polymer technologies such as ePTFE. With the growth of TAVR, one of the strongest arguments in favor of PHVs is their unique application for transcatheter delivery—namely the ability of polymer leaflets to be crimped to very small calibers with supposedly reduced microstructure disruption, though this remains to be tested in larger scale studies. Importantly, a significant challenge that remains for the field of PHVs is successfully demonstrating long-term *in vivo* durability. While few technologies have undergone large animal testing, only the Foldax Tria surgical valve has been tested in a human trial, though small and of limited duration. We suspect this will be the focus of the next phase of PHV research and development and will dictate the future role that PHVs will play in the clinical landscape of valve therapy.

## Author contributions

SS and MK: literature review, drafting of the manuscript, creation of figures and tables, and revisions. EC, YX, DK, and GF: reviewing and editing of the manuscript. IG: drafting of manuscript and reviewing and editing of the manuscript. All authors contributed to the article and approved the submitted version.

## Funding

SS received funding *via* the Thoracic Surgery Foundation Resident Research Award. GF received from NIH grant R01HL143008, Berrie Pre-Translational Diabetes Research Award, The Kibel Fund for Aortic Valve Research, The Valley Hospital Foundation “Marjorie C Bunnel” charitable fund, and Andrew Sabin Family Foundation Cardiovascular Research Laboratory.

## Conflict of interest

IG reports the following disclosures: consultant (honoraria)—Zimmer Biomet, Atricure, Neosurgery, Neptune Medical, Abbvie, Johnson & Johnson, Boston Scientific, Edwards Lifesciences, Medtronic. Advisory Boards: Edwards Surgical, Medtronic Surgical, Trisol Medical, Abbvie, Johnson & Johnson, Foldax Medical, Zimmer Biomet, Neosurgery, Abbvie, Boston Scientific.Equity: Valcare Medical, Durvena, CardioMech, Vdyne, MitreMedical, MITRx.Institutional funding to Columbia University: Edwards Lifesciences, Medtronic, Abbott Vascular, Boston Scientific, JenaValve.

The remaining authors declare that the research was conducted in the absence of any commercial or financial relationships that could be construed as a potential conflict of interest.

## Publisher’s note

All claims expressed in this article are solely those of the authors and do not necessarily represent those of their affiliated organizations, or those of the publisher, the editors and the reviewers. Any product that may be evaluated in this article, or claim that may be made by its manufacturer, is not guaranteed or endorsed by the publisher.
